# Between No Help and Coercion: Toward Referral to Involuntary Psychiatric Admission. A Qualitative Interview Study of Stakeholders' Perspectives

**DOI:** 10.3389/fpsyt.2021.708175

**Published:** 2021-08-06

**Authors:** Irene Wormdahl, Tonje Lossius Husum, Solveig Helene Høymork Kjus, Jorun Rugkåsa, Trond Hatling, Marit B. Rise

**Affiliations:** ^1^NTNU Social Research, Norwegian Resource Centre for Community Mental Health, Trondheim, Norway; ^2^Department of Mental Health, Faculty of Medicine and Health Sciences, Norwegian University of Science and Technology, Trondheim, Norway; ^3^Centre for Medical Ethics, Institute for Health and Society, University of Oslo, Oslo, Norway; ^4^Faculty of Health Sciences, Oslo Metropolitan University, Oslo, Norway; ^5^Health Service Research Unit, Akershus University Hospital, Lørenskog, Norway; ^6^Centre for Care Research, University of South-Eastern Norway, Porsgrunn, Norway

**Keywords:** involuntary admission, severe mental illness, mental health services, primary mental health care, mental health, psychiatry

## Abstract

**Objective:** Paths toward referral to involuntary psychiatric admission mainly unfold in the contexts where people live their everyday lives. Modern health services are organized such that primary health care services are often those who provide long-term follow-up for people with severe mental illness and who serve as gatekeepers to involuntary admissions at the secondary care level. However, most efforts to reduce involuntary admissions have been directed toward the secondary health care level; interventions at the primary care level are sparse. To adapt effective measures for this care level, a better understanding is needed of the contextual characteristics surrounding individuals' paths ending in referrals for involuntary admission. This study aims to explore what characterizes such paths, based on the personal experiences of multiple stakeholders.

**Method:** One hundred and three participants from five Norwegian municipalities participated in individual interviews or focus groups. They included professionals from the primary and secondary care levels and people with lived experience of severe mental illness and/or involuntary admission and carers. Data was subject to constant comparison in inductive analysis inspired by grounded theory.

**Results:** Four main categories emerged from the analysis: deterioration and deprivation, difficult to get help, insufficient adaptation of services provided, and when things get acute. Combined, these illustrate typical characteristics of paths toward referral for involuntary psychiatric admission.

**Conclusion:** The results demonstrate the complexity of individuals' paths toward referral to involuntary psychiatric admission and underline the importance of comprehensive and individualized approaches to reduce involuntary admissions. Furthermore, the findings indicate a gap in current practice between the policies to reduce involuntary admissions and the provision of, access to, and adaptation of less restrictive services for adults with severe mental illness at risk of involuntary admissions. To address this gap, further research is needed on effective measures and interventions at the primary care level.

## Introduction

Involuntary psychiatric admissions go against the fundamental health care principle of patient autonomy (1, 2). Many individuals exposed to such admissions, along with their carers, report experiences of fear and distress (3, 4). Moreover, evidence that coercive practices lead to better outcomes is limited (5–7). Despite international and national policies to reduce the use of involuntary admissions in mental health, numbers indicate that rates of this practice have increased in several European countries (8). This is cause for growing concern, and less restrictive alternatives and effective measures in mental health services to prevent involuntary admissions are called for (9).

Reported rates of involuntary admissions vary (8). In Italy and Austria, the rates per 100,000 people in 2015 was 14.5 and 282, respectively (8). Norway reports relatively high numbers, with a rate of 186 per 100,000 persons 16 years and older in 2018 (10). Different clinical and social factors have been associated with an increased risk of involuntary admission; a recent review found that a psychotic disorder, previous involuntary hospitalization, lack of adherence to medication, police involvement in admission process, male gender, being unemployed, living on welfare, being single, limited social support, and living in deprived areas are associated with higher risk of involuntary admission (11).

In most Western countries, care for people with severe and long-lasting mental illness has moved from psychiatric hospitals to communities, where individuals' paths toward referral to involuntary admissions unfold within complex contexts, often with the involvement of multiple stakeholders (12). Although services at the primary health care level play a key role in providing services to people with severe mental illness (SMI), the role of these services in such paths remains largely undocumented (13, 14). Persons with lived experience of involuntary admission have reported lack of information and involvement in treatment decisions (3); carers experience difficulties getting preventive help prior to an individual's acute crisis and report lack of adequate support for themselves in such situations (4). A systematic review and meta-synthesis of multiple stakeholders' experiences with involuntary psychiatric admission decision-making found that collaboration between the services involved was lacking, that medical views dominated assessments, and that the admission process could be experienced as heavy-handed (especially given that it often involves police) (15). Previous studies have largely been limited to experiences during detention, of the admission process, and of the admission decision-making process (3, 4, 15). In the Norwegian context, studies focusing on the involuntary admission process have primarily been quantitative (16). Thus, studies incorporating multiple stakeholders' experiences in earlier phases of individuals' paths, including how primary health services are involved and interact, can contribute to understanding how to target further development of services at this care level so as to reduce involuntary admissions. Therefore, this study aimed to explore multiple stakeholders' experiences with paths toward referral to involuntary psychiatric admission.

## Materials and Methods

### Study Setting

The current study is part of a cluster randomized controlled trial that sought to develop and test a primary care-level intervention aimed at reducing involuntary psychiatric admissions (ClinicalTrials.gov, NCT03989765). Ten mid-sized Norwegian municipalities with twenty to fifty thousand inhabitants participated. The associated regional psychiatric hospitals and their community mental health centers from the secondary care level, who serve the municipalities' populations, were also involved. The municipalities receiving intervention took part in developing and testing the intervention. To prepare for this, comprehensive mapping of current practices was conducted using qualitative methods, and the analysis presented here is based on these data. In the following, involuntary psychiatric admissions are those sanctioned by the Norwegian Mental Health Care Act § 3-2 (involuntary observation) and § 3-3 (involuntary admission) (17).

In Norway mental health care is provided by two separate health care levels: primary and secondary level. Primary mental health care, often organized jointly with primary addiction services, is provided by the 356 municipalities. Among other things, it includes supportive housing (with or without resident staff), day-care facilities, home care, therapeutic conversations, and leisure activities. General practitioners (GPs) are organized at the primary health care level. This includes both the GPs (family doctors) and primary medical emergency services. In addition, social care, (un)employment services, municipal housing, and local police are among the services provided by the municipalities.

The power to subject people to involuntary admission is held by services at the secondary mental health care level. At this level, regional psychiatric hospitals and community mental health centers provide specialist inpatient and outpatient treatment, including community-based ambulant treatment. Norwegian mental health legislation sets out stringent criteria for involuntary admissions, requiring that options for voluntary engagement have been exhausted (17). It is also required that the need for involuntary admission is assessed by a medical practitioner outside of the secondary inpatient unit (unless the individual is under a community treatment order). Thus, referral to involuntary psychiatric admission is usually carried out by a primary care-level GP. The GP conducts a medical assessment of the need for a referral. If an individual refuses the assessment, the chief municipal medical officer has the authority to decide on an involuntary medical examination. When a person is referred, the individual and the referral are dispatched to secondary care, typically the acute inpatient hospital unit.

### Participants and Recruitment

This study involved 103 participants, including multiple stakeholders from the five intervention municipalities who had experienced or been involved in individuals' paths to involuntary admissions. Eligible participants were: (1) people currently working in various services and who had experience supporting individuals who had been involuntarily admitted; (2) people with lived experience of SMI and/or involuntary admission; and (3) carers of individuals with lived experience of SMI and/or involuntary admission. The stakeholder services represented were primary mental health services, chief municipal medical officers, GPs, medical emergency services, police, outpatient specialist mental health services, and inpatient specialist mental health services. Eligible participants in primary and secondary services were recruited through service managers; GPs were recruited through the chief municipal medical officers. People with lived experience were recruited through the local groups of the advocacy organization Mental Health Norway, and carers were recruited through the local groups of the advocacy organizations Mental Health Norway and Mental Health Carers Norway. Purposive sampling was used to obtain a sample with a wide range of participants representing multiple stakeholders. See Table 1 for sample description.

**Table 1:** Distribution of participants.

**Variable**	**Informants** **(*N* = 103)**	**Percent**
**Sex**		
Male	43	42
Female	60	58
**Role/service**		
Primary mental health services	32	31
Secondary mental health services	16	16
Primary medical services^*^	16	16
Police	2	2
People with lived experience	16	16
Carers	21	20
**Level of education among participants working in services (** ***n*** **=** **66)**		
Vocational education training	1	2
3 years higher professional education	9	13
>3 years higher professional education	56	85
**Municipality**		
Municipality 1	29	28
Municipality 2	13	13
Municipality 3	22	21
Municipality 4	17	17
Municipality 5	22	21
**Type of interview**		
Individual interviews^**^	68	66
Focus groups	35	34

* *Primary medical services include general practitioners (GPs), medical emergency services (GPs and nurses), and chief municipal officers*.

** *Three were conducted as group interviews with four, two, and two participants, respectively*.

### Data Collection

A mix of individual interviews and focus groups were conducted. For the most part, professionals were interviewed individually, and people with lived experiences and carers participated in focus groups. In the focus groups, the participants' joint experiences could be utilized but not to the same degree as an individual focus, given that the theme of the study involved possibly traumatic personal experiences for participants with lived experience and for carers.

A total of 60 individual interviews were conducted. Upon request, three interviews of professionals were conducted as group interviews with four, two, and two participants, respectively (*n* = 8). Seven focus groups were conducted (*n* = 35). The focus groups had between two and seven participants. Two interviews with carers were conducted as individual interviews because we did not manage to recruit more people in their municipalities. All interviews in one municipality were conducted before we moved on to the next municipality. Data collection was conducted in the period June 2019 to December 2019.

The interviews were based on a semi-structured interview guide. In individual interviews, the participants working in different services were asked to describe one or two of the most recent situations they had been directly or indirectly involved in that ended up with someone being involuntarily admitted. The interviewer probed for contextual information and the sequence of events, including who was involved and how. People with lived experience and carers were asked to describe paths to involuntary admissions more generally, not necessarily about their personal experiences (although several of them chose to talk about this). Examples of questions included what they believed are typical circumstances leading up to an involuntary admission, who could be involved and how, what services individuals commonly receive prior to an involuntary admission, what happens in situations where an individual is referred to involuntary admission, and how services collaborate with the individual and their carers. All interviewees were also asked if they were aware of the rate of involuntary admissions in their municipality. Other themes in the semi-structured interview guide, such as factors in current practice that can affect pathways to involuntary admissions, and suggested measures to prevent such admissions, are and will be published elsewhere (17).

All interviews were conducted face-to-face except four that were conducted by phone. IW and TLH jointly carried out the interviews with the participants working in different services in the first municipality, then worked separately in two municipalities each. The interviews lasted 25–80 min and were conducted in meeting rooms in the municipality's offices. Upon request, two interviews were conducted at the participants' home. The focus groups and individual interviews with people with lived experience and with carers were carried out by IW, TLH and SHHK jointly in the first municipality, then in pairs; SHHK worked in all municipalities, and IW and TLH worked in two municipalities each. The focus groups lasted 90–110 min and were conducted in meeting rooms at the primary mental health services' location or on the premises of the local groups of the respective advocacy organizations.

### Data Analysis

The analysis was inspired by grounded theory (18). The inductive analysis resulted in a conceptualized model revealing the characteristics of individuals' paths toward referral to involuntary admission.

In the first analytic step, IW, TLH, and SHHK wrote comprehensive notes during the interviews and focus groups. In the focus groups, we also logged our perceptions of the group dynamics. After a day of interviews, the notes were immediately used to write condensed summaries of the interviews. These condensed summaries were then used to write a reflection memo, including the interviewers' preliminary analyses of the participants' experiences. The first reflection memo was written after the first seven interviews of primary mental health professionals in one municipality; for the focus groups, reflection memos were written after each group session. In this phase, we included characteristics seen in single interviews as well as patterns across interviews. Throughout the interview period, the reflection memos were regularly subjected to constant comparison. Typically, this was performed within the scopes of participants in the same stakeholder group and municipalities. As we moved from one municipality to the next, new characteristics evolved and merged into categories, which were subjects for further exploration in new interviews. In the second analytic step, after all the interviews were completed, IW, TLH, and SHHK read the overall condensed summaries and reflection notes. During this process, characteristics were merged and rearranged, and categories were reviewed. In the third analytic step, all authors participated in further analysis. Preliminary categories and characteristics were reviewed several times until consensus was reached. In the final step, we prepared quotes from the data material to illustrate and elaborate the results. These quotes are non-verbatim condensations of the participants' descriptions.

### Ethics

The Regional Committees for Medical and Health Research Ethics in Norway (REC) considered the study outside their remit (REC reference number 2018/2382 C), and the study was approved by the Norwegian Centre for Research Data (NSD reference number 743586). Informed written consent in accordance with the General Data Protection Regulation (GDPR) was obtained from all participants. No names or personal identification information were registered in the condensed summaries or reflection memos from the interviews. Information about users, participants and services in the condensed summaries presented as examples of situations are anonymized and kept to a minimum to ensure anonymity.

## Results

**Figure 1:**
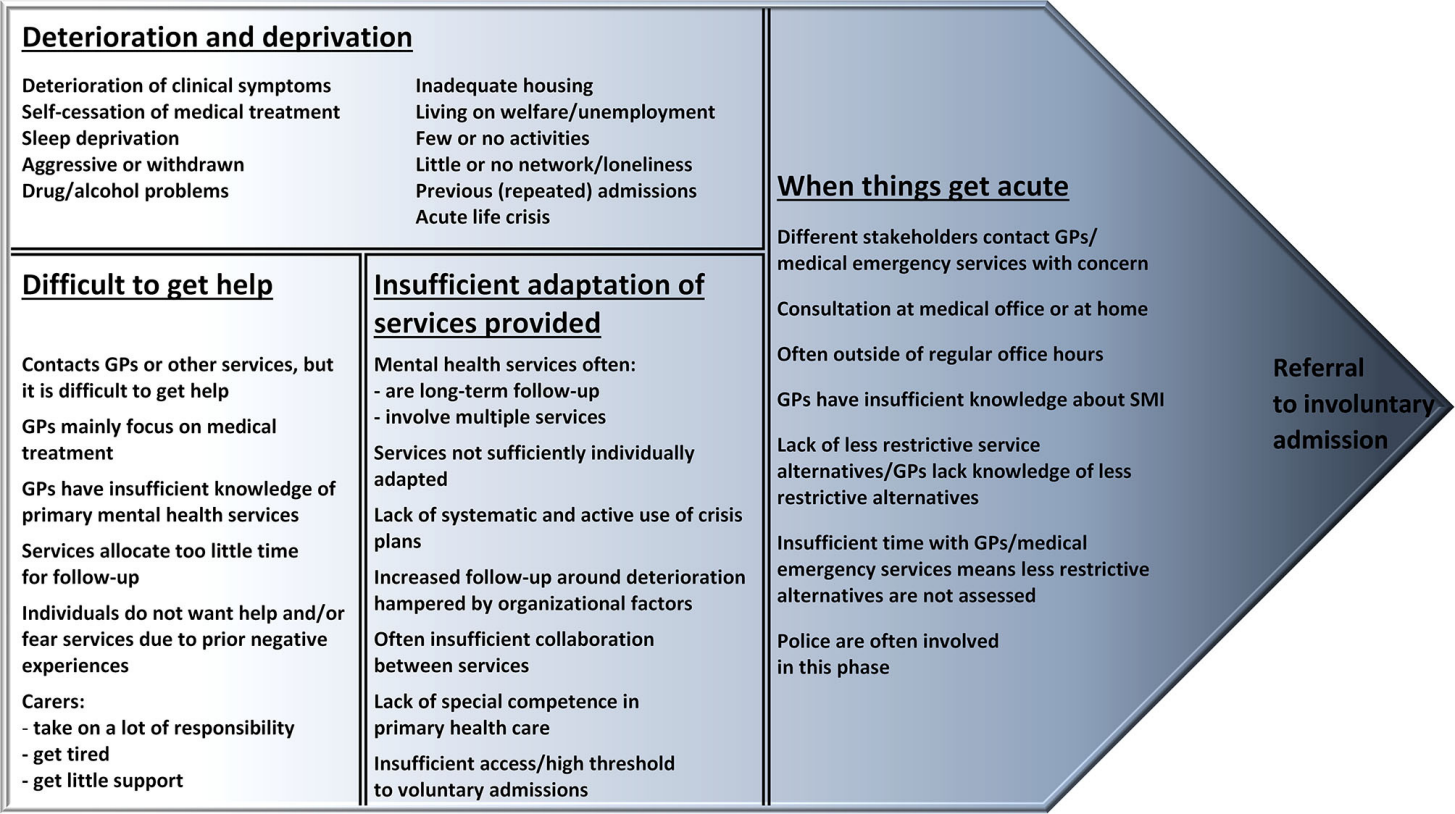
Between no help and coercion: Toward referral to involuntary psychiatric admission.

Drawing on the experiences of multiple stakeholders in five Norwegian municipalities, the analyses identified four main categories: deterioration and deprivation, difficult to get help, insufficient adaptation of services provided, and when things get acute. In Figure 1, the model “*Between no help and coercion: Toward referral to involuntary psychiatric admission”* displays the categories and their characteristics. Although the mutual ending point is referral to involuntary admission, the categories in the model are not necessarily sequential. For instance, an individual's path could comprise characteristics from two, three, or all four categories. Moreover, various characteristics could apply at different times for different people, and some were present throughout an individual's path. Furthermore, some described an unexpected acute life crisis that caused deterioration of clinical symptoms without the presence of other characteristics in the category *deterioration and deprivation*; these situations quickly moved on to the category *when things get acute* without including other characteristics shown in the two other categories. Nevertheless, the majority described multiple characteristics that were present before the severity of mental health deterioration was said to be acute, extending the paths' timeline and often including characteristics from several categories.

### Deterioration and Deprivation

As seen in Figure 1, the category *deterioration and deprivation* indicate that a variety of clinical symptoms, behavioral symptoms, and socioeconomic factors were present in individuals' paths toward referral to involuntary admission. Examples of clinical symptoms described were psychosis, suicidality, self-harm, drug addiction, comprehensive trauma history (PTSD), cognitive impairment, and severe depression. In addition, many linked self-cessation of psychotropic medication and sleep deprivation with deterioration of clinical symptoms. Both reserved/withdrawn behavior and aggressive behavior were mentioned as typical symptoms, with the latter being the dominating characteristic of described paths.

*This man lived in a municipal apartment related to a supported housing service with day-care staff. Prior to his last involuntary admission, we understood a deterioration was in progress when he withdrew more and more. Usually when he got like this, he had stopped taking his medication without anybody noticing. He would not let us in when we came to see him, and we had to persuade him to talk to us. For a while he let himself be persuaded to let us in, and we could motivate him to eat and go for a walk with us. But as he kept on not taking his medication, he deteriorated more and more. This is a man with a massive trauma history, and gradually he appeared more and more psychotic, until the situation became acute. At that point, he had not slept for several days, probably not eaten a lot either, and he started acting out, appeared aggressive, and threatened mental health staff that tried to get in contact with him*. (Staff, primary mental health service)

Many explained that these characteristics had been present over time, while a few talked about acute appearance of clinical and behavioral symptoms due to an acute life crisis.

*My sister had always had a seemingly well-functioning life with a husband, kids, house, car, and a dog. But when her husband filed for divorce, she did not cope well. She went into an acute major depression and tried to kill herself*. (Carer, sibling)

Participants described how many individuals who were subjected to referral to involuntary admission ended up as “revolving door patients,” with repeated involuntary admissions. Some professionals knew of individuals who had been involuntarily admitted as much as 50–100 times in 1 year.

*He goes out on the edge to jump in front of the train and says he will kill himself. This happens again and again and again. When he stands there someone from the public calls 911, the police and ambulance turn up, get him down from the bridge, and the police drive him to the medical emergency service, who then refers him to involuntary admission. After a short stay at the acute ward of the psychiatric hospital he gets discharged, usually within a few days. Last year I think he did this over 50 times!* (Staff, primary mental health service)

People with these kinds of vulnerabilities were also described as being exposed to combinations of the sociodemographic vulnerabilities shown in *deterioration and deprivation* in Figure 1. This was believed to increase the likelihood of entering a path ending in a referral to involuntary admission. Participants described individuals living in a variety of contexts: some lived in private accommodations, others in municipal housing, supported housing with milieu staff, or had no fixed residence. Some lived with family members, others lived alone. Many participants observed that inadequate living conditions were prevalent; they described individuals with unstable housing situations, reduced capability to manage residential living, and poorly adapted municipal housing facilities. For instance, municipal housing contexts where people felt unsafe were mentioned as a barrier to recovery for individuals with SMI.

*It is not ideal for him to live in that municipal building downtown where everybody else also has severe problems. People with all kinds of problems live there, and he gets anxious when the neighbors act out or make noise. In addition, he is not too good at comprehending and interpreting others when they communicate; he often misunderstands and gets into conflict with neighbors. In his worse periods, all this can enhance his deterioration and make it difficult for him to regain good daily functioning. I think he should have lived in another place where he could retire and protect himself a bit more from people who don't serve him well*. (Staff, primary mental health service)

Lack of suitable employment opportunities or meaningful daily activities were described as resulting in inactivity and passivity. In addition, many individuals were described as having little or no social network, which combined with inactivity often led to loneliness. Participants with lived experience and carers especially emphasized loneliness, and many experienced that the stigma around SMI in the wider society heightened the individuals' loneliness; several participants said the cares became the only social network for the individual. Employment, meaningful activities, and personal networks were emphasized as factors that, when present, could facilitate personal recovery and could prevent deterioration and the risk of referral to involuntary admission.

*There is too much focus on just illness and too little focus on the fact that life consists of more than just illness. You need to have a place to live, practical help, and things that can make life a bit easier. I think a more diverse offer of activities to those who need it would be good, because there is not much to choose from now, especially for men. We have a day center but they offer mostly knitting, crocheting and reading the newspaper and stuff like that. They should organise things like data, golf, bowling and outdoor activities. It is time for some innovation. It is important to have good arenas to meet, generally in the community, in the city, or where you live, but the municipality here has no other activities to offer outside the day center*. (Individual with lived experience)

### Difficult to Get Help

As seen in Figure 1, the category *difficult to get help* indicates how many participants experienced that insufficient support was available for at-risk individuals in the early phases of illness development. In their experience, the process often started before services got involved. Several participants with lived experience and carers described how they had tried to contact both GPs and other primary services several times in this phase of an individual's path, without receiving adequate help.

*But I think there is something wrong with the system when they did not notice my signals earlier because I did not become psychotic overnight. Looking back, I think that it was not difficult to see the signals. When I did not dare going to the pharmacy or did not go to work back then when I was working, then the signals are visible. It is strange that they could not react earlier to my deterioration. Then, perhaps, I just needed a little more follow-up than once a week over a period of time. And that my GP, the mental health service, and my employer could collaborate a little more. I see that it would cost something, but I think that socio-economically it must be cheaper than me ending up being involuntary admitted. Maybe if I had gotten help earlier the total cost would be less, and my symptoms would be milder and quality of life better*. (Individual with lived experience)

*She already started to get ill when she dropped out of high school, almost a year before the involuntary admission. She isolated herself, withdrew from her friends, and kind of changed personalities. We suspected that she had started doing drugs. We tried to get help, both through school and her GP, but no one seemed to understand how severe it was. And when, several months later, she finally got some follow-up from the mental health and addiction team in the municipality, she had become so severely ill with psychosis and all that it did not help. There must be something more between coercion and absolutely nothing*. (Carer, parent)

Many participants with lived experience and carers said that GPs often relied on medication as the main treatment option for people with SMI. In addition, participants from all stakeholder groups, including GPs, mentioned that GPs had limited knowledge of the available low-threshold services in primary mental health care. Several participants with lived experience and carers stated that GPs did not have sufficient time to conduct comprehensive assessments of their needs and match them with available services. This was also mentioned in relation to other services, such as when specialist outpatient mental health services only allocated a 1-h follow-up each week; according to participants with lived experience and carers, this was insufficient to help someone with SMI who deteriorated.

*I felt that we did not get help fast enough when the crisis appeared. It was like there was nothing between no help and coercion. My wife had to become very, very, ill before they understood the severity of her condition, and then it ended in an involuntary admission. I believe that if the doctor had taken better time to hear us out and gotten more insight into her problems, she could have gotten better help and recovery before she got so ill that she had to be involuntarily admitted*. (Carer, spouse)

Some participants from the primary mental health services described how some individuals with SMI refused to receive mental health services in the periods prior to or between involuntary admissions. In these situations, the professionals felt that there was little they could do until the individual became so ill that a referral to involuntary admission was necessary. They described how they had limited opportunities to work more thoroughly with individuals unless their time allocations and work-load were reorganized to allow extra effort to prevent further deterioration. At the same time, participants with lived experience and carers discussed how some individuals with SMI withdrew from services because they had experienced former admissions as traumatic. Among other things, they talked about being roughly handled, and often the police had been involved. When this happened in public, the participants experienced additional strain and stigma. Some said that the services were not tailored to help people overcome this fear around receiving services.

When people did not receive sufficient support, carers felt that they had to take a lot of responsibility for their loved ones. Many said this was stressful at times, and they could get exhausted as their loved ones' mental health deteriorated. According to the carers, there was little, if any, service approach or support for them as carers to help them manage these situations.

### Insufficient Adaptation of Services Provided

As seen in Figure 1, the category *insufficient adaptation of services provided* represents characteristics emphasizing how the provision of essential services for people with SMI was not sufficiently tailored to individuals' needs. Many individuals who received mental health services prior to a referral to involuntary admission had done so for long periods, often years. Some received multiple services, and from both the primary and secondary care levels. Nonetheless, many participants experienced that the long-term follow-up from mental health services, both prior to and during their path toward referral to involuntary admission, often lacked the degree of continuity people with SMI needed.

Professional participants from primary mental health services said they sometimes tried preventive measures when an individual showed early signs of deterioration. For instance, when an individual receives ambulant services, some professionals said they could try to increase follow-up and come by the individual's home several times a week in critical periods. However, several participants felt that this flexibility was hampered by the lack of resources, service organization, and knowledge. A few professional participants said that people with SMI at risk of involuntary admissions had crisis plans that guided the measures to implement, although the majority explained that the use of crisis plans varied greatly and were often neither established nor actively used. Poor collaboration among services, both at the primary level and between the primary and secondary levels, was highlighted by participants in all stakeholder groups as affecting adaptation of services. Many of the professionals working in mental health services experienced difficulties in collaboration with GPs, and collaboration between primary and secondary mental health services was also often experienced as poor or absent. Many participants mentioned that good collaboration depended on the people and was not part of a systemic approach.

*Many services were involved—our service [primary mental health service], the activity center, an outpatient psychologist from the secondary mental health service, and the GP. In addition, his carers were there. But in my experience, the services did not collaborate much. One service did not know what another service did. The help provided was concurrent and not complementary, and coordination between the primary and secondary services were lacking. To my perception, a general lack of clarity in the division of tasks and responsibilities in this municipality is not good for individuals with severe mental illness, who often need multiple services and individual adaptation*. (Staff, primary mental health service)

Many professionals from primary mental health services said they lacked the right competence and tools to divert individuals' paths from ending in referral to involuntary admission. This was echoed by many GPs and professionals from secondary services.

*I am not sure if this is a group of people that the primary mental health services are capable of handling. At least, it appears like they have too little knowledge in how to handle aggression and agitation, and it also seems like the ones working there get anxious in situations like that. Then the working routine might end up with the staff calling the police as soon as the slightest indication of conflict appears, instead of being able to help them calm down. And you know, in a supportive housing there can be many situations that potentially can cause conflicts, like shortage of cigarettes, money, or a drugged neighbour frightening you*. (Staff, secondary mental health service)

Furthermore, several participants revealed insufficient access to voluntary inpatient treatment at a secondary mental health care facility prior to an individual's deterioration becoming so severe that referral to involuntary admission was deemed necessary. In the experience of several GPs and primary mental health professionals, the threshold for people to be voluntarily admitted at secondary mental health inpatient services was often too high. A lack of beds (capacity) at the secondary care level was mentioned as a possible explanation.

*When I really needed and wanted to be admitted, it was rejected. And I know how sick I can get! But it just did not happen! Psychiatry is a very rigid system*. (Individual with lived experience)

### When Things Get Acute

As seen in Figure 1, the category *when things get acute* represents characteristics experienced as being present when the deterioration has become so severe that a referral to involuntary admission is on the cards. People from four stakeholder groups were typically the ones who contacted GPs to express their concern: (1) carers, private network, or others from the community; (2) professionals working in primary health services; (3) the police; and (4) professionals working in secondary mental health services. Sometimes the individual's GP received the concern, but in most cases those with a concern contacted the primary emergency medical service. Occasionally, carers directed their concern to the chief municipal medical officer; this mostly involved “revolving door patients,” where the individual and carers knew the chief municipal officer from previous admissions. Chief municipal officers were also contacted by others from the community when they were concerned for an individual's mental health; examples of these were neighbors, animal welfare inspectors, and the fire brigade. In addition, some participants said that carers could express their concern with a primary mental health service if the individual received follow-up from this service. In situations where individuals with SMI did not give any response or let anyone in, GPs had to contact the chief municipal officer, who could decide on an involuntary medical examination.

*We heard about the concern through a friend of the woman when she had not collected her mail for a while. The women did not answer the phone and did not open the door when we went to her home to make contact. This was a woman we had known for a long time, and we knew she had a severe mental illness. We contacted her GP, who contacted the chief municipal officer, who decided on an involuntary medical examination. The GP called on the police to break into the woman's home. There, we—the police, GP, and me—found her in bed in a state where she appeared to be very psychotic. She denied that she was ill and refused to be admitted. The GP referred her to involuntary admission, and the police had to escort her by force out to their car to drive her to the hospital. I think that when the police need to be involved in these situations, it makes the situation appear very dramatic. I think about how it must appear to the neighbors or others passing by*. (Staff, primary mental health service)

The participants shared that sometimes the police were the first ones in contact with people in acute situations. This could occur when an individual acted out in public or stood on a bridge and seemed prepared to commit suicide. In these situations, the participants said the police were the ones who brought the individual to the GP for medical assessment. According to the police participants, they were mostly involved in such cases outside their regular office hours. They said they did not know who to call in other services when they were faced with an acute psychiatric crisis. Consequently, the medical emergency service became the primary available place where they shared their concern. In the participants' experience, a few referrals to involuntary admissions occurred solely within secondary mental health care; typically, this happened if an individual was placed under a community treatment order. In these situations, the participants explained that professionals from outpatient secondary mental health services could direct their concern directly to their inpatient unit, and involvement from others besides secondary mental health services and police could be absent.

When a GP conducted a medical assessment to see whether a referral was warranted, they typically performed it either at the GP's office or in the individual's home. However, most referrals were described as happening outside of regular office hours. As a result, assessments were often conducted by GPs at emergency medical services. This meant that the doctor conducting the assessment had no or limited knowledge of the individual involved. Participants from all stakeholder groups expressed that, in their experience, the GPs had inadequate knowledge of SMI. Furthermore, they felt that GPs lacked knowledge about less restrictive alternatives at the primary care level. As mentioned in the category *insufficient adaptation of services provided*, professional participants also said they often did not have an available, updated crisis plan that could guide them on which measures to implement. Consequently, involuntary admission became the only option considered in acute situations.

*When an individual comes to the medical emergency service with mental health and addiction problems, I often just refer them to the secondary services. I do not contact the primary services because I simply do not know enough about them*. (GP, emergency medical service)

In addition, several GPs said that other service alternatives were limited in an acute situation. For instance, primary mental health services were not available outside of regular office hours. In addition, the services could have intake time that was incompatible with the acuteness of the situation, and characteristic symptoms for this target group often prevented them from using the acute inpatient beds in primary health care. Another circumstance said to make involuntary admission the “only” option was that medical practitioners had too little time for individual consultations.

*The medical emergency service has to take it all! We are the only service that has to deal with all kinds of problems and illnesses, arrange it all, fix it all. Often it is hectic and time is limited, and we quickly have to find a solution for a critical situation. At that point, the easiest alternative is to refer people further into the health system, and in these situations, this means referring them to involuntary admission at the [name of the acute ward at the secondary mental health inpatient unit]. The police, carers, or those who have brought the individual to the medical emergency service, typically at night or on the weekends, also want us to request an involuntary admission. They stand here waiting, and at the same time many others with different problems and illnesses are waiting too. So, the quickest and simplest solution might be to send them with the police to [name of acute ward]. Referral to involuntary admission often becomes the solution, because significantly longer time is needed to find any primary services that could be an alternative*. (GP, emergency medical service)

Also, participants with lived experience mentioned that GPs often had to little time at consultations.

*GPs have to little time. It is important that the GP takes his time when meeting us. It is important that they know you. If you are heard and understood you can get more appropriate help*. (Individual with lived experience)

Several participants perceived that the police were often involved in one way or another in this phase. Participants said the police were typically called to assist with transport if an individual was assessed as aggressive, violent, or affected by noticeable intoxication, or if they had to break into an individual's home to enable access for health staff. The approach of the police varied. For instance, some described how the police used rough methods when breaking into homes or used force when escorting individuals from public places. Others had experienced the police's approach as caring and helpful, and they described how the police took their time and talked with the individual or let the individual perform their morning routine or put on make-up before they were brought away.

## Discussion

A conceptualized model based on multiple stakeholders' experiences and displaying categories and characteristics of individuals' paths toward referral to involuntary admission was developed from the results (Figure 1). Typically, multiple characteristics were present, and most paths started prior to the acute situation that resulted in a referral to involuntary admission. The clinical and socioeconomic characteristics described by participants in the current study generally match what is known in the literature as factors associated with involuntary admissions (11, 16). Consequently, the discussion will instead focus on some of the shortages in service provision appearing within such trajectories, including difficulties in accessing and adapting services, insufficient assessment of possibilities to use, and lack of less restrictive service alternatives, before implications for practice are highlighted.

“*It was like there was nothing between no help and coercion”* was a statement that is illustrative of many carers' experiences. Both individuals with lived experience and carers said it was difficult to get help in an early phase of an individual's mental health deterioration. Many GPs had a medical focus and lacked knowledge of primary mental health services. Individuals with SMI who received long-term mental health services often experienced service limitations, inadequate individual adaptation, and limited possibilities to act upon individuals' shifting needs. In addition, insufficient collaboration between services was revealed. Jankovic et al. (19) also found that carers perceived that services responded to crises rather than prevented them. This implies a potential to prevent some involuntary admissions, if services can be provided at an earlier stage of an individual's deterioration. Potential may be found especially in the phase where people make contact with their GP or other services asking for help. “*There is too much focus on just illness and too little focus on the fact that life consists of more than just illness”* was a statement from a participant with lived experience. If a medical perspective dominates, assessment of the individual's overall situation might be limited, and thus access to services that focus more on personal and social recovery in an early phase of deterioration will not be provided. Furthermore, factors that can hamper individual service adaptation include limited resources within services, budget cuts, rigid allocation systems, heavy caseloads, no or arbitrary use of crisis plans, and limited opportunities for voluntary admissions prior to the situation becoming acute (17, 20). Lack of alternatives due to a narrow range of housing, activity, and employment opportunities for people with SMI can negatively affect personal recovery and services' ability to reverse individuals' paths toward referral to involuntary admission (12, 17, 21).

Reducing referrals to involuntary admission cannot be taken separately from the provision of other services. The results in the current study indicate that acute situations are characterized by a lack of less restrictive care alternatives. Furthermore, even when potential alternatives were present, they were not always assessed as an option. For instance, busy medical emergency services with no or limited knowledge of the individual led GPs to choose referral to involuntary admissions instead of taking on the more time-consuming work of arranging other alternatives. In addition, the GPs' knowledge of current primary mental health services that provide alternatives were perceived as insufficient. Mental health legislation requires that options for voluntary engagement have been exhausted (22); therefore, it is important to question whether some individuals might experience unlawful referrals to involuntary admissions, if services for this group are organized in such a way that the time-consuming nature of considering voluntary alternatives in an acute situation sometimes becomes the rationale for referral to involuntary admission. A recent review of initiatives to reduce coercion in mental health clearly state that facilitating voluntary support requires a range of community services from which service users can choose (23). Unavailability of less restrictive care alternatives has been found to predict decisions of referral to involuntary admission (24, 25). In fact, a lack of alternatives has been found to be more significant than mental disorder, dangerousness, or individuals' refusal of care (24). In Norway, a discrepancy between referrals to involuntary admission and psychiatrists' decisions to involuntarily admit have been seen in about one-third of the cases (10, 16). This strengthens the notion of insufficient provision of or access to less restrictive service alternatives for this target group.

Furthermore, these paths might bring about ethical challenges for the professionals involved. In situations where professionals must choose between an individual's right to autonomy and their right to health care (26) when less restrictive alternatives are lacking, the health care organization influences this choice. Professionals are then left with a different ethical dilemma: to choose between involuntary admission and neglect. Consequently, involuntary admission might become the only moral choice to safeguard the individual, and the rationale and justification of the involuntary admission are potentially left unchallenged. Floyd (27) found that most professionals were comfortable or totally comfortable with cases they had handled wherein people were involuntarily admitted. This indicates that provision of less restrictive service alternatives, and services' capability to adapt according to individuals' shifting needs, might be influenced by mental health professionals' attitudes toward involuntary admissions. In Norway, the health government's directives to reduce involuntary admissions have formally addressed the secondary health care level (28). Without this being on the agenda at all care levels, professionals at the primary care level might simply continue their former ways of doing things (15), leaving involuntary admissions unquestioned with regard to the organization and provision of services between care levels. A lack of systematic focus in primary mental health services on reducing involuntary admissions (17) might indicate that professionals' attitudes toward involuntary admissions have not been particularly challenged at this care level.

### Strengths and Limitations

The results of the current study represent the experiences of multiple stakeholders in five Norwegian municipalities. Thus, they may not be representative elsewhere. However, the high number of participants from several municipalities and from multiple stakeholder groups strengthens the possibility of generalization across settings. Including multiple stakeholders and stakeholder groups moderated personification and strengthened external validity. The results represent the participants' experiences with individuals' paths toward referral to involuntary admission and are limited to situations that end with such a referral (and, subsequently, an involuntary admission). The focus groups recruited through the advocacy organization Mental Health Norway included both participants with lived experience and carers. This might have limited disagreements in the discussions between these stakeholder groups. This study was part of a larger project that sought to develop and test an intervention at the primary mental health care level, aiming to reduce the use of involuntary admissions; this could have affected the experiences and examples the participants shared, potentially making them more inclined to describe cases where they thought referral to involuntary admission could have been avoided. However, our impression was that we obtained a mix of different experiences, including those where participants perceived that such referrals could not have been avoided. A multidisciplinary research group with three researchers (including a peer researcher) performing interviews, and an additional extended research group participating in the analysis process, strengthen the internal validity of the results.

### Implications for Practice and Research

The conceptual model “*Between no help and coercion: Toward referral to involuntary psychiatric admission”* developed in this study indicates a gap in current practice between, on the one hand, the policies to reduce involuntary admissions and, on the other hand, the provision of, access to, and individual adaptation of less restrictive service alternatives for adults with SMI at risk of referral to involuntary admission. Given these perspectives, we recommend that further service development and research aim to facilitate:

- Easy access to services in early phases of deterioration.
- Individualized adaptation of service provision, housing, and activities.
- Systematic use of joint crisis plans.
- Enough consultation time and flexibility in service provision.
- Collaboration among services facilitating complementary and comprehensive treatment and care.
- Knowledge in primary health care on SMI, involuntary admissions, and alternatives to involuntary admissions.
- Access to less restrictive service alternatives in acute situations.

## Conclusion

The aim of this study was to explore the characteristics of the paths toward referral to involuntary psychiatric admission of adults with SMI. Based on the personal experiences of multiple stakeholders in five Norwegian municipalities, the four main categories of deterioration and deprivation, difficult to get help, insufficient adaptation of services provided, and when things get acute are illustrated in a conceptual model displaying the characteristics of such paths. The model demonstrates the complexity of individuals' paths and underlines the importance of comprehensive approaches, along with the flexibility to tailor service delivery to individual needs, in working to prevent involuntary admissions. Furthermore, the results in this study indicate a gap in current practice between, on the one hand, the policies to reduce involuntary admissions and, on the other hand, access to, adaptation of, and provision of less restrictive services for adults with SMI at risk of involuntary admission. Further research is needed on effective measures and interventions at the primary care level.

## Data Availability Statement

Data sharing is not applicable for the datasets generated in this study due to their containing information that could compromise the privacy of research participants, further inquiries can be directed to the corresponding author.

## Author Contributions

IW, TLH, SHHK, and TH developed the interview guides, recruited participants, conducted the interviews, and performed constant comparison and preliminary analyses. SHHK had a particular focus on the experiences of participants with lived experience and of carers. The writing of the manuscript was led by IW. TH, SHHK, JR, TLH, and MBR participated in the critical review of several drafts. All authors participated in final analyses and discussions of how the results were related to existing literature, contributed to planning the study, and revised and approved the final manuscript.

## Author Disclaimer

The views expressed are those of the authors and not the Council.

## Conflict of Interest

The authors declare that the research was conducted in the absence of any commercial or financial relationships that could be construed as a potential conflict of interest.

## Publisher's Note

All claims expressed in this article are solely those of the authors and do not necessarily represent those of their affiliated organizations, or those of the publisher, the editors and the reviewers. Any product that may be evaluated in this article, or claim that may be made by its manufacturer, is not guaranteed or endorsed by the publisher.

## Abbreviations

SMI: severe mental illness
GP: general practitioner
PTSD: posttraumatic stress disorder.
